# Insights into Australian Bat Lyssavirus in Insectivorous Bats of Western Australia

**DOI:** 10.3390/tropicalmed4010046

**Published:** 2019-03-11

**Authors:** Diana Prada, Victoria Boyd, Michelle Baker, Bethany Jackson, Mark O’Dea

**Affiliations:** 1 School of Veterinary Medicine, Murdoch University, Perth, WA 6150, Australia; b.jackson@murdoch.edu.au (B.J.); m.odea@murdoch.edu.au (M.O.); 2 Australian Animal Health Laboratory, CSIRO, Geelong, VIC 3220, Australia; vicky.boyd@csiro.au (V.B.); michelle.baker@csiro.au (M.B.)

**Keywords:** Australian bat lyssavirus, microbats, Western Australia, serology, Luminex, real-time PCR

## Abstract

Australian bat lyssavirus (ABLV) is a known causative agent of neurological disease in bats, humans and horses. It has been isolated from four species of pteropid bats and a single microbat species (*Saccolaimus flaviventris*). To date, ABLV surveillance has primarily been passive, with active surveillance concentrating on eastern and northern Australian bat populations. As a result, there is scant regional ABLV information for large areas of the country. To better inform the local public health risks associated with human-bat interactions, this study describes the lyssavirus prevalence in microbat communities in the South West Botanical Province of Western Australia. We used targeted real-time PCR assays to detect viral RNA shedding in 839 oral swabs representing 12 species of microbats, which were sampled over two consecutive summers spanning 2016–2018. Additionally, we tested 649 serum samples via Luminex^®^ assay for reactivity to lyssavirus antigens. Active lyssavirus infection was not detected in any of the samples. Lyssavirus antibodies were detected in 19 individuals across six species, with a crude prevalence of 2.9% (95% CI: 1.8–4.5%) over the two years. In addition, we present the first records of lyssavirus exposure in two *Nyctophilus* species, and *Falsistrellus* *mackenziei*.

## 1. Introduction

Australian bat lyssavirus (ABLV) is one of the 16 classified species of lyssaviruses within the family *Rhabdoviridae* [1]. It was first discovered in Australia in 1996 [2] and early studies distinguished two variants, the pteropid variant carried by all four species of flying fox within continental Australia [3], and the insectivorous variant detected only in the yellow-bellied sheath-tailed bat (*Saccolaimus flaviventris*) [4]. Although there is evidence of ABLV exposure in 11 genera within four microbat families [5], additional reservoir species have yet to be identified.

Both ABLV strains are associated with clinical disease in the host species [2,4]. Although spillover events are extremely rare, they have resulted in fatal neurological disease in humans and horses [6,7,8], making ABLV an agent of significant public health concern. Current public health policy recommends a prophylactic rabies vaccination for bat handlers, with the administration of post-exposure treatment including vaccination and rabies immunoglobulin based on vaccination history and individual immune status [9]. However, the perceived risk from exposure to microbats is potentially limited by the relative lack of media exposure these species receive compared to the larger pteropids, coupled with only a single documented microbat to human transmission of ABLV to date.

Since the discovery of ABLV, surveillance has predominantly relied on passive sampling regimes, with a single published study based on active sampling in the east and north of the country [10]. Results suggest ABLV circulates at a low prevalence (<1%) in healthy wild bat populations [5]. However, the prevalence (and therefore risk) escalates to 5%–10% where bats are injured, sick or orphaned. These are precisely the conditions which are considered to drive human exposure through rescue and rehabilitation attempts, or the protection of property, pets or children [11,12,13]. Interestingly, the public health research on ABLV and human-bat interactions to date does not report on community knowledge or risk perceptions of microbats versus pteropid species, generally referring to ‘bats’ as a broad group [11,12,13,14,15]. Therefore, whilst basic knowledge of ABLV in bats appears to be high in some regions [11], public awareness of the specific risks and recommended post-exposure behaviours with respect to microbats warrants further research.

In Western Australia, ABLV surveillance has also been sporadic and passive, with the only targeted study focusing on the far northern part of the state and concentrating mainly on the pteropid bats in the region [5], with sample collection occurring some 15 years ago. Active surveillance of microbats has likely been hindered by the additional time and resource demands of sampling non-cave roosting species typical of the region [16]. Therefore, there is limited current information on the ABLV status of Western Australian microbat species and the disease status is assumed by the extrapolation of information from bat populations in the eastern states. Additionally, there is no local data of ABLV status in the south west of Western Australia, an area with arguably increased human-bat interaction due to the higher population density.

In order to better inform the regional risks associated with human-bat interactions, this study aimed to establish the lyssavirus status of insectivorous bats in the South-West Botanical Province of WA over a period of two years. We used an ABLV specific and a pan-lyssavirus reverse transcription real-time PCR (RRT-PCR) assay to screen oral swabs from 12 species of microbats. Additionally, we used a bead-based Luminex^®^ assay on serum samples to determine previous lyssavirus exposure.

## 2. Materials and Methods 

All sampling was approved by the Department of Parks and Wildlife of Western Australia, permits 08-001359-1, and CE005517. Capture, handling and sampling procedures were approved by the Murdoch University Animal Ethics Committee (R2882/16).

Harp traps and mist nets were used to capture bats at different locations of the South-West Botanical Province (SWBP), an Australian global biodiversity hotspot. The province covers approximately 44 million hectares and comprises nine bioregions [17]. Remnant natural cover in the east and west of the region is separated by the extensive monoculture known as the Western Australian wheatbelt, which acts as a major dispersion barrier for many native species.

**Figure 1:**
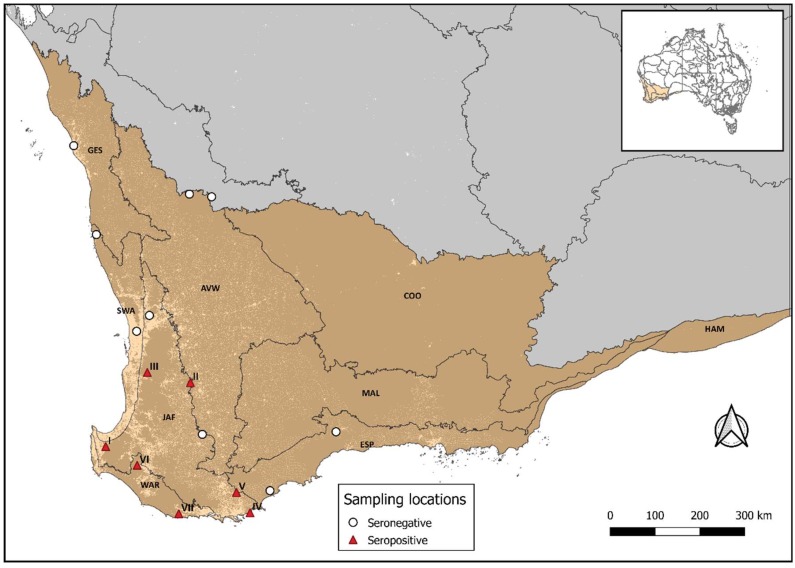
The South West Botanical province (SWBP) highlighted in brown. Sampling sites are shown and sites where seropositive individuals were identified are labelled I-VII. Presence (light areas) and absence (dark areas) of human populations are shown. The SWBP encompasses nine bioregions, Avon Wheatbelt (AVW), Coolgardie (COO), Esperance Plains (ESP), Geraldton Sandplains (GES), Hampton (HAM), Jarrah Forest (JAF), Mallee (MAL), Swan Coastal Plain (SWA), and Warren (WAR).

Sampling took place over two summers between 2016 and 2018, with sites in the east and west boundaries of the wheatbelt. The northeastern sites were within the semi-arid Avon bioregion which was predominantly sampled during the first season (2016–2017). The southwestern sites were distributed across five bioregions, the Esperance Plains, Geraldton Sandplains, Jarrah Forest, Swan Coastal Plain, and Warren (Figure 1), which were mainly sampled during the second year of the study (2017–2018). Therefore, most sampling sites were visited only once over the two years, except for locations in the Avon bioregion which were sampled during both summers.

Prior to undertaking trapping, all personnel involved in handling bats underwent a complete rabies vaccination schedule. Biosecurity and biosafety protocols during handling and sampling included the use of protective gloves while manipulating bats out of traps and nets. During sample collection, double gloves were worn while restraining the animal (nitrile gloves over protective gloves), with the nitrile gloves changed between each bat. All surfaces and non-disposable equipment (e.g., calipers) were disinfected with a 10% solution of F10 (Health and Hygiene, South Africa) between each bat. Additionally, single calico bags were used for each individual and soaked in F10 before being re-used.

Following capture, bats were taxonomically identified, and a single oral swab (FLOQSwab, Copan, Brescia, Italy) was collected per individual and stored in RNAlater^®^ (Ambion, Life Technologies, Carlsbad, CA, USA). Additionally, 10 µL of blood was taken from the brachial vein and diluted 1:10 in phosphate buffered saline (PBS). 

Oral swabs were vortexed and 50 µL of the supernatant used as starting material for all extractions using a Magmax viral RNA extraction kit (Ambion, Applied Biosystems, Vilnius, Lithuania) following the manufacturer’s instructions. The detection of lyssavirus RNA was performed using an Australian bat lyssavirus RRT-PCR specific for the insectivorous variant of the virus [18], and a pan-lyssavirus RRT-PCR assay [19]. Inactivated insectivorous ABLV RNA provided by the CSIRO Australian Animal Health Laboratory (AAHL, Victoria, Australia) was used as a positive control. Assays were performed on a QuantStudio 6 Flex platform (Life Technologies, Singapore). Nucleic acid extraction verification and lack of inhibitors were assessed using an endogenous 18S rRNA PCR assay (Life Technologies, Pleasanton, CA, USA). 

Sera were tested for reactivity to lyssavirus antigens [20] in an indirect binding Luminex^®^ assay [21], at a final working dilution of 1:50 at CSIRO AAHL. As a pilot study, samples collected during the first season were pooled one in three (*n* = 246) or one in four (*n* = 24). All samples collected during the second season were tested individually (*n* = 391). Median Fluorescence Intensity (MFI) was read using a Bio-Plex instrument (Bio-Rad Laboratories, Hercules, CA, USA). Due to the lack of known positive and known negative bat sera from the species captured in this study to validate the assay, the MFI threshold to differentiate positive and negative samples was set at 1000 MFI as per CSIRO protocols. Previous studies published by the Australian Animal Health Laboratory and elsewhere using the same Bio-Plex platform have used a threshold of at least three times the mean MFI of negative sera from other bat species with values below 250 MFI considered negative [22,23,24,25]. The same principle was used here to establish a threshold based on an MFI of 250 corresponding to a negative sample with sample MFIs above 1000 considered positive.

Prevalence estimates and 95% confidence intervals were calculated using the Wilson’s Method [26] as implemented in the R package *epitools* [27]. 

## 3. Results

**Figure 2:**
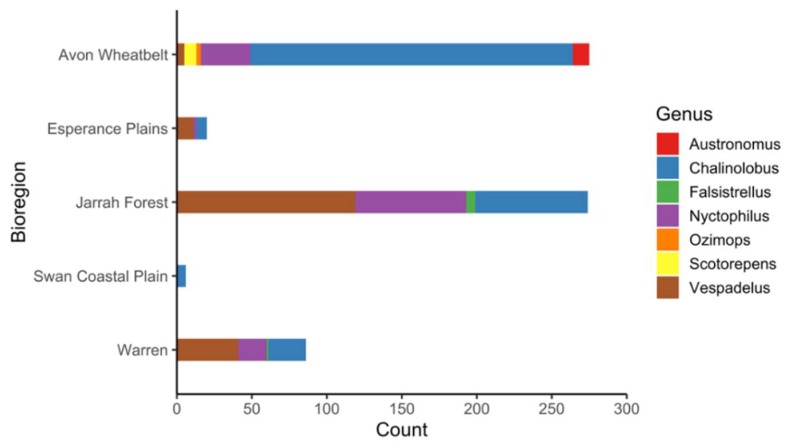
Number of blood samples taken by genera and bioregion. Note, no blood samples were obtained from bats in the Geraldton Sandplains bioregion.

In total, 839 oral swabs and 661 blood samples were collected. Twelve samples did not provide a valid Luminex^®^ assay result and were removed from the analysis. Therefore, the final serological dataset comprised 649 samples encompassing 12 bat species (Table 1). Captured species composition varied at each location (Figure 2) and in general *Chalinolobus gouldii* and *Vespadelus regulus* had the greatest representation in the swab and sera data sets (Table 1). A total of 270 serum samples were collected in the first year and 379 during the second year. 

Neither the ABLV specific or the pan-lyssavirus RRT-PCR reactions yielded a positive result. No inhibition was detected in any of the samples and positive and negative controls were valid. 

Serological reactivity to lyssavirus antigens was detected in 19 samples (Table 1, Table S1) resulting in an overall antibody prevalence of 2.9% (95% CI: 1.8–4.5%). Seropositive samples encompassed six species, *V. regulus* had the highest prevalence at 5.5% (95% CI: 2.9–10.1%), followed by *C. morio* (4.6%, 95% CI: 1.6–12.8), *Nyctophilus gouldi* (4.5%, 95% CI: 1.5–12.5) and *C. gouldii* (0.7%, 95% CI: 0.2–2.7%). Additionally, reactivity was also detected in a single *Falsistrellus mackenziei*, and an *N. major*. Due to their small sample sizes, prevalence values were not estimated for these two species (Table 1).

Despite a large number of individuals being sampled during the first year (*n* = 270), none reacted on the lyssavirus serological assay, thus all seropositive bats were captured during the second year of the study, and south of the wheatbelt. Consequently, annual seroprevalence significantly increased from 0% in the first year to 5% (95% CI: 3.2–7.7%) in the second year (*p* = < 0.0001). It should be noted that the majority (78%) of captures in the first year and north of the wheatbelt were dominated by a single species, *C. gouldii*. 

Seropositive individuals were captured at seven sites which were dominated by natural jarrah (*Eucalyptus marginata)* and marri (*Corymbia calophylla)* forests, and on one occasion on agricultural land transitioning to wandoo (*E. wandoo*) woodlands (Table 2). Seropositive *V. regulus* were sampled at five different locations, seropositive *N. gouldii*, and *C. morio* were captured at three and two locations, respectively, and the remaining two species were all captured at a single site. When examining the data by number of seropositive samples per location, three different species were captured at three locations, and the remaining four locations were represented by a single seropositive species.

## 4. Discussion

Here we report the first comprehensive study investigating the lyssavirus status of apparently healthy populations of insectivorous bats in the South West Botanical Province of WA. Significantly, this is the first active surveillance to take place in the SWBP and therefore constitutes an update to the existing limited lyssavirus data on wild microbat populations [10]. We did not detect any current ABLV infection or the presence of any other lyssavirus species circulating within the sampled populations, despite the large sample size (*n* = 839). The suitability of oral swabs for lyssavirus detection has previously been demonstrated in clinical studies [18,28,29,30], and in active lyssavirus surveillance efforts [31,32]. Nonetheless, diagnostic sensitivity in a field surveillance setting for the detection of ABLV may be limited by the combination of intermittent shedding [33], and a small window of infection [32,34] of an already low prevalence virus. 

Serological results indicated previous lyssavirus exposure in 19 individuals, resulting in an overall seroprevalence of 2.9% in the study population. This result is congruent with previous sero-response estimates of wild Australian bat populations [5], albeit using a different serological assay. Antibody reactivity was detected in six Vespertilionidae species, *C. gouldii, C. morio, V. regulus, F. mackenziei*, *N. gouldi*, and *N. major*. The presence of lyssavirus antibodies has previously been documented for the *Chalinolobus* and *Vespadelus* genera [5], and the results here constitute the first published report of lyssavirus exposure in *Nyctophilus spp*. and *F. mackenziei,* an endemic species of the jarrah forests of the South West. It was not possible to carry out further confirmatory testing on seropositive individuals, as the volume of blood ethically permissible to be drawn from microbats was fully used in the Luminex^®^ assay. 

Seroprevalence values per species varied between 0.7% and 5.5%, with the highest values observed in *V. regulus* (5.5%), *C. morio* (4.6%) and *N. gouldi* (4.5%). Despite the existence of previously published seroprevalence figures for some Australian Vespertilionidae species [5], it is not appropriate to make direct comparisons due to previous results grouping different species by genera and being based on an alternative serological assay. Even though seropositivity for ABLV has previously been reported in *Austronomus australis* [5], we did not detect any antibody response in this species, possibly due to the small sample size (*n* = 11). 

All seropositive individuals originated from seven locations within the Jarrah and Warren bioregions and none from the Avon bioregion, which is isolated by the Australian wheatbelt. It is unclear whether this partitioning of the data represents a geographical, temporal or a species association given these risk factors are confounded in the study population. However, temporal shedding may explain the 0% prevalence in the Avon bioregion despite the large sample size (*n* = 226), as this region was predominantly sampled in the first year and all the seropositive individuals occurred in the second year of the study. This hypothesis is supported by longitudinal studies elsewhere showing that inter-annual variation of seroprevalence estimates is common in wild bat populations [28,32]. Further, long term active surveillance in *Myotis myotis* has provided important insights into the infection dynamics of lyssavirus at a temporal and geographical scale [32], highlighting how similar studies would contribute to better understanding of ABLV dynamics within Australian microbat populations. It is possible that a failure to detect seropositive samples during the first year could also have been the result of decreased assay sensitivity triggered by sample pooling. However, titration studies using pools of *Pteropus alecto* sera by CSIRO AAHL have not demonstrated a loss of assay sensitivity at the pooling levels used in this study.

The detection of ABLV seropositivity in the SWBP, particularly the southern parts of the region, further supports the existence of additional lyssavirus reservoirs, as the distribution of *Pteropus spp.* and *Saccolaimus flaviventris* does not extend to the SWBP. This suggests that any hypothetical reservoir may be a member of the families Vespertilionidae or Molossidae, with serology results indicating Vespertilionidae may be a reservoir genus in the Southwest of WA. Importantly, seropositive individuals of six separate species came from a variety of areas in the south west in relatively close proximity to towns and recreational areas (<20 km). 

The results from this study suggest that active infections in wild microbat populations may be even lower than previously thought. Despite this, the evidence of circulating ABLV in the region validates current recommendations for post-exposure treatment of people with bat bites and scratches, including those from microbats. We recommend public health research akin to that conducted in the eastern states [11,12,13,14,15] in order to evaluate knowledge, attitudes and perceptions of ABLV risks in the south west of WA specifically for microbat species. This information should be used to guide future public health campaigns in the region. 

## Figures and Tables

**Table 1:** Seroprevalence of Australian Bat Lyssavirus in 12 species of microbats of the South West Botanical Province of Western Australia. The total number of samples tested and positives () are shown.

Family	Species	Swabs	Sera	Seroprevalence ^1^
Vespertilionidae	*Chalinolobus gouldii*	287(0)	262(2)	0.7 (0.2–2.7)
	*Chalinolobus morio*	105(0)	64(3)	4.6 (1.6–12.8)
	*Falsistrellus mackenziei*	14(0)	7(1)	NC ^2^
	*Nyctophilus geoffroyi*	69(0)	48(0)	
	*Nyctophilus gouldi*	78(0)	66(3)	4.5 (1.5–12.5)
	*Nyctophilus major*	12(0)	5(1)	NC ^2^
	*Nyctophilus sp* ^3^	6(0)	5(0)	
	*Scotorepens balstoni*	13(0)	8(0)	
	*Vespadelus baverstocki*	6(0)	5(0)	
	*Vespadelus finlaysoni*	1(0)	0	
	*Vespadelus regulus*	227(0)	164(9)	5.5 (2.9–10.1)
	*Vespadelus sp* ^3^	2(0)	1(0)	
Molossidae	*Austronomus australis*	13(0)	11(0)	
	*Ozimops sp*	6(0)	3(0)	

^1^ Prevalence (%) and 95% confidence intervals (CI). ^2^ Prevalence estimates not calculated (NC) due to small sample size. ^3^ These individuals were not confidently identified to species level, however will belong to either of the listed *Nyctophilus* or *Vespadelus* species, and therefore do not count towards the total number of species sampled.

**Table 2:** Distribution of seropositive individuals per bioregion and trapping location. Number of positives () per species.

Bioregion	Location	Species
Jarrah Forest	I	*Vespadelus regulus* (1)
	II	*Chalinolobus gouldii* (2)
	III	*Vespadelus regulus* (2)
		*Nyctophilus major* (1)
		*Falsistrellus mackenziei* (1)
	IV	*Nyctophilus gouldi* (1)
	V	*Vespadelus regulus* (1)
Warren	VI	*Chalinolobus morio* (2)
		*Nyctophilus gouldi* (1)
		*Vespadelus regulus* (1)
	VII	*Chalinolobus morio* (1)
		*Nyctophilus gouldi* (1)
		*Vespadelus regulus* (4)

## Supplementary Materials

The following are available online at https://www.mdpi.com/2414-6366/4/1/46/s1, Table S1 Median Fluoresce Intensity values for all sera tested.
