# Supplementary material for: Insights into Australian Bat Lyssavirus in Insectivorous Bats of Western Australia
Source: Trop Med Infect Dis. 2019 Mar 11;4(1):46. doi: 10.3390/tropicalmed4010046 (PMC6473685; doi:10.3390/tropicalmed4010046)
Supplement: Supplementary file 1 [file tropicalmed-04-00046-s001.pdf]

Table S1. Median Fluorescence Intensity (MFI) values obtained from the bead based Lumines assay. Samples from the first season were pooled one in three or one in four. Therefore the MFI value represents an aggregate estimate for those samples. Samples above 1000 MFI are shown in bold.

Season	Bioregion	Species				MFI
1	Avon Wheatbealt	Chalinolobus gouldii	Chalinolobus gouldii	Chalinolobus gouldii		84
1	Avon Wheatbealt	Chalinolobus gouldii	Chalinolobus gouldii	Chalinolobus gouldii		91
1	Avon Wheatbealt	Chalinolobus gouldii	Chalinolobus gouldii	Chalinolobus gouldii		93
1	Avon Wheatbealt	Chalinolobus gouldii	Chalinolobus gouldii	Chalinolobus gouldii		95
1	Avon Wheatbealt	Chalinolobus gouldii	Chalinolobus gouldii	Chalinolobus gouldii		98
1	Avon Wheatbealt	Chalinolobus gouldii	Chalinolobus gouldii	Chalinolobus gouldii		104
1	Avon Wheatbealt	Chalinolobus gouldii	Chalinolobus gouldii	Chalinolobus gouldii		107
1	Avon Wheatbealt	Chalinolobus gouldii	Chalinolobus gouldii	Chalinolobus gouldii		107
1	Avon Wheatbealt	Chalinolobus gouldii	Chalinolobus gouldii	Chalinolobus gouldii		129
1	Avon Wheatbealt	Chalinolobus gouldii	Chalinolobus gouldii	Chalinolobus gouldii		150
1	Avon Wheatbealt	Chalinolobus gouldii	Chalinolobus gouldii	Chalinolobus gouldii		161
1	Avon Wheatbealt	Chalinolobus gouldii	Chalinolobus gouldii	Chalinolobus gouldii		169
1	Avon Wheatbealt	Chalinolobus gouldii	Chalinolobus gouldii	Chalinolobus morio		77
1	Avon Wheatbealt	Chalinolobus gouldii	Chalinolobus gouldii	Chalinolobus morio		96
1	Avon Wheatbealt	Chalinolobus gouldii	Chalinolobus gouldii	Chalinolobus morio		99
1	Avon Wheatbealt	Chalinolobus gouldii	Chalinolobus gouldii	Chalinolobus morio		123
1	Avon Wheatbealt	Chalinolobus gouldii	Chalinolobus gouldii	Nyctophilus geoffroyi		74
1	Avon Wheatbealt	Chalinolobus gouldii	Chalinolobus gouldii	Nyctophilus geoffroyi		76
1	Avon Wheatbealt	Chalinolobus gouldii	Chalinolobus gouldii	Nyctophilus geoffroyi		80
1	Avon Wheatbealt	Chalinolobus gouldii	Chalinolobus gouldii	Nyctophilus geoffroyi		86
1	Avon Wheatbealt	Chalinolobus gouldii	Chalinolobus gouldii	Nyctophilus geoffroyi		88
1	Avon Wheatbealt	Chalinolobus gouldii	Chalinolobus gouldii	Nyctophilus geoffroyi		91
1	Avon Wheatbealt	Chalinolobus gouldii	Chalinolobus gouldii	Nyctophilus geoffroyi		91
1	Avon Wheatbealt	Chalinolobus gouldii	Chalinolobus gouldii	Nyctophilus geoffroyi		94
1	Avon Wheatbealt	Chalinolobus gouldii	Chalinolobus gouldii	Nyctophilus geoffroyi		96
1	Avon Wheatbealt	Chalinolobus gouldii	Chalinolobus gouldii	Nyctophilus geoffroyi		97
1	Avon Wheatbealt	Chalinolobus gouldii	Chalinolobus gouldii	Nyctophilus geoffroyi		102
1	Avon Wheatbealt	Chalinolobus gouldii	Chalinolobus gouldii	Nyctophilus geoffroyi		104
1	Avon Wheatbealt	Chalinolobus gouldii	Chalinolobus gouldii	Nyctophilus geoffroyi		105
1	Avon Wheatbealt	Chalinolobus gouldii	Chalinolobus gouldii	Nyctophilus geoffroyi		108
1	Avon Wheatbealt	Chalinolobus gouldii	Chalinolobus gouldii	Nyctophilus geoffroyi		125
1	Avon Wheatbealt	Chalinolobus gouldii	Chalinolobus gouldii	Nyctophilus geoffroyi		130
1	Avon Wheatbealt	Chalinolobus gouldii	Chalinolobus gouldii	Nyctophilus geoffroyi		132
1	Avon Wheatbealt	Chalinolobus gouldii	Chalinolobus gouldii	Nyctophilus major		127
1	Avon Wheatbealt	Chalinolobus gouldii	Chalinolobus gouldii	Nyctophilus sp		105
1	Avon Wheatbealt	Chalinolobus gouldii	Chalinolobus gouldii	Ozimops sp		108
1	Avon Wheatbealt	Chalinolobus gouldii	Chalinolobus gouldii	Ozimops sp		120
1	Avon Wheatbealt	Chalinolobus gouldii	Chalinolobus gouldii	Scotorepens balstoni		0
1	Avon Wheatbealt	Chalinolobus gouldii	Chalinolobus gouldii	Scotorepens balstoni		83
1	Avon Wheatbealt	Chalinolobus gouldii	Chalinolobus gouldii	Scotorepens balstoni		95
1	Avon Wheatbealt	Chalinolobus gouldii	Chalinolobus gouldii	Scotorepens balstoni		98
1	Avon Wheatbealt	Chalinolobus gouldii	Chalinolobus gouldii	Scotorepens balstoni		105
1	Avon Wheatbealt	Chalinolobus gouldii	Chalinolobus gouldii	Scotorepens balstoni		107
1	Avon Wheatbealt	Chalinolobus gouldii	Chalinolobus gouldii	Scotorepens balstoni		112
1	Avon Wheatbealt	Chalinolobus gouldii	Chalinolobus gouldii	Scotorepens balstoni		133
1	Avon Wheatbealt	Chalinolobus gouldii	Chalinolobus gouldii	Vespadelus baverstocki		81
1	Avon Wheatbealt	Chalinolobus gouldii	Chalinolobus gouldii	Vespadelus baverstocki		106
1	Avon Wheatbealt	Chalinolobus gouldii	Chalinolobus gouldii	Vespadelus baverstocki		108
1	Avon Wheatbealt	Chalinolobus gouldii	Chalinolobus gouldii	Vespadelus baverstocki		174
1	Avon Wheatbealt	Chalinolobus gouldii	Chalinolobus gouldii	Vespadelus baverstocki		243
1	Avon Wheatbealt / Jarrah Forest	Chalinolobus gouldii	Chalinolobus gouldii	Chalinolobus gouldii*	Chalinolobus gouldii*	108
1	Avon Wheatbealt / Jarrah Forest	Chalinolobus gouldii	Chalinolobus gouldii	Chalinolobus gouldii*	Chalinolobus gouldii*	109
1	Avon Wheatbealt / Jarrah Forest	Chalinolobus gouldii	Chalinolobus gouldii	Chalinolobus gouldii*	Chalinolobus gouldii*	136
1	Avon Wheatbealt / Jarrah Forest	Chalinolobus gouldii	Chalinolobus gouldii	Chalinolobus gouldii*		75
1	Avon Wheatbealt / Jarrah Forest	Chalinolobus gouldii	Chalinolobus gouldii	Chalinolobus gouldii*		85
1	Avon Wheatbealt / Jarrah Forest	Chalinolobus gouldii	Chalinolobus gouldii	Chalinolobus gouldii*		97
1	Avon Wheatbealt / Jarrah Forest	Chalinolobus gouldii	Chalinolobus gouldii	Chalinolobus gouldii*		105
1	Avon Wheatbealt / Jarrah Forest	Chalinolobus gouldii	Chalinolobus gouldii	Chalinolobus gouldii*		115
1	Avon Wheatbealt / Jarrah Forest	Chalinolobus gouldii	Chalinolobus gouldii	Chalinolobus gouldii*		120
1	Avon Wheatbealt / Jarrah Forest	Chalinolobus gouldii	Chalinolobus gouldii	Chalinolobus morio*		196
1	Avon Wheatbealt / Jarrah Forest	Chalinolobus gouldii	Chalinolobus gouldii	Nyctophilus geoffroyi*		76
1	Avon Wheatbealt / Jarrah Forest	Chalinolobus gouldii	Chalinolobus gouldii	Nyctophilus geoffroyi*		80
1	Avon Wheatbealt / Jarrah Forest	Chalinolobus gouldii	Chalinolobus gouldii	Nyctophilus geoffroyi*		89
1	Avon Wheatbealt / Jarrah Forest	Chalinolobus gouldii	Chalinolobus gouldii	Nyctophilus geoffroyi*		107

1	Avon Wheatbealt / Jarrah Forest	Chalinolobus gouldii	Chalinolobus gouldii	Nyctophilus geoffroyi*		110
1	Avon Wheatbealt / Jarrah Forest	Chalinolobus gouldii	Chalinolobus gouldii	Nyctophilus geoffroyi*		111
1	Avon Wheatbealt / Jarrah Forest	Chalinolobus gouldii	Chalinolobus gouldii	Nyctophilus geoffroyi*		113
1	Avon Wheatbealt / Jarrah Forest	Chalinolobus gouldii	Chalinolobus gouldii	Nyctophilus geoffroyi*		141
1	Avon Wheatbealt / Jarrah Forest	Chalinolobus gouldii	Chalinolobus gouldii	Nyctophilus gouldi*		103
1	Avon Wheatbealt / Jarrah Forest	Chalinolobus gouldii	Chalinolobus gouldii	Vespadelus regulus*	Vespadelus regulus*	94
1	Avon Wheatbealt / Jarrah Forest	Chalinolobus gouldii	Chalinolobus gouldii	Vespadelus regulus*	Vespadelus regulus*	96
1	Avon Wheatbealt / Jarrah Forest	Chalinolobus gouldii	Chalinolobus gouldii	Vespadelus regulus*	Vespadelus regulus*	126
1	Avon Wheatbealt / Jarrah Forest	Chalinolobus gouldii	Chalinolobus gouldii	Vespadelus regulus*		69
1	Avon Wheatbealt / Jarrah Forest	Chalinolobus gouldii	Chalinolobus gouldii	Vespadelus regulus*		81
1	Avon Wheatbealt / Jarrah Forest	Chalinolobus gouldii	Chalinolobus gouldii	Vespadelus regulus*		93
1	Avon Wheatbealt / Jarrah Forest	Chalinolobus gouldii	Chalinolobus gouldii	Vespadelus regulus*		96
1	Avon Wheatbealt / Jarrah Forest	Chalinolobus gouldii	Chalinolobus gouldii	Vespadelus regulus*		101
1	Avon Wheatbealt / Jarrah Forest	Chalinolobus gouldii	Chalinolobus gouldii	Vespadelus regulus*		105
1	Avon Wheatbealt / Jarrah Forest	Chalinolobus gouldii	Chalinolobus gouldii	Vespadelus regulus*		116
1	Avon Wheatbealt / Jarrah Forest	Chalinolobus gouldii	Chalinolobus gouldii	Vespadelus regulus*		132
1	Avon Wheatbealt / Jarrah Forest	Chalinolobus gouldii	Chalinolobus gouldii	Vespadelus regulus*		150
1	Avon Wheatbealt / Jarrah Forest	Chalinolobus gouldii	Chalinolobus gouldii	Vespadelus regulus*		210
1	Avon Wheatbealt / Jarrah Forest	Chalinolobus gouldii	Chalinolobus gouldii	Vespadelus regulus*		123
1	Avon Wheatbealt / Swan Coastal Plain	Chalinolobus gouldii	Chalinolobus gouldii	Chalinolobus gouldii*		78
1	Avon Wheatbealt / Swan Coastal Plain	Chalinolobus gouldii	Chalinolobus gouldii	Chalinolobus gouldii*		86
1	Avon Wheatbealt / Swan Coastal Plain	Chalinolobus gouldii	Chalinolobus gouldii	Chalinolobus gouldii*		103
1	Avon Wheatbealt / Swan Coastal Plain	Chalinolobus gouldii	Chalinolobus gouldii	Chalinolobus gouldii*		105
1	Avon Wheatbealt / Swan Coastal Plain	Chalinolobus gouldii	Chalinolobus gouldii	Chalinolobus gouldii*		119
2	Avon Wheatbelt	Austronomus australis				112
2	Avon Wheatbelt	Austronomus australis				127
2	Avon Wheatbelt	Austronomus australis				129
2	Avon Wheatbelt	Austronomus australis				145
2	Avon Wheatbelt	Austronomus australis				148
2	Avon Wheatbelt	Austronomus australis				150
2	Avon Wheatbelt	Austronomus australis				156
2	Avon Wheatbelt	Austronomus australis				193
2	Avon Wheatbelt	Austronomus australis				279
2	Avon Wheatbelt	Austronomus australis				400
2	Avon Wheatbelt	Austronomus australis				463
2	Avon Wheatbelt	Chalinolobus gouldii				77
2	Avon Wheatbelt	Chalinolobus gouldii				103
2	Avon Wheatbelt	Chalinolobus gouldii				104
2	Avon Wheatbelt	Chalinolobus gouldii				106
2	Avon Wheatbelt	Chalinolobus gouldii				117
2	Avon Wheatbelt	Chalinolobus gouldii				122
2	Avon Wheatbelt	Chalinolobus gouldii				123
2	Avon Wheatbelt	Chalinolobus gouldii				127
2	Avon Wheatbelt	Chalinolobus gouldii				129
2	Avon Wheatbelt	Chalinolobus gouldii				131
2	Avon Wheatbelt	Chalinolobus gouldii				134
2	Avon Wheatbelt	Chalinolobus gouldii				140
2	Avon Wheatbelt	Chalinolobus gouldii				145
2	Avon Wheatbelt	Chalinolobus gouldii				145
2	Avon Wheatbelt	Chalinolobus gouldii				146
2	Avon Wheatbelt	Chalinolobus gouldii				152
2	Avon Wheatbelt	Chalinolobus gouldii				156
2	Avon Wheatbelt	Chalinolobus gouldii				163
2	Avon Wheatbelt	Chalinolobus gouldii				164
2	Avon Wheatbelt	Chalinolobus gouldii				168
2	Avon Wheatbelt	Chalinolobus gouldii				185
2	Avon Wheatbelt	Chalinolobus gouldii				186
2	Avon Wheatbelt	Chalinolobus gouldii				199
2	Avon Wheatbelt	Nyctophilus geoffroyi				112
2	Avon Wheatbelt	Nyctophilus geoffroyi				112
2	Avon Wheatbelt	Nyctophilus geoffroyi				114
2	Avon Wheatbelt	Nyctophilus geoffroyi				127
2	Avon Wheatbelt	Nyctophilus geoffroyi				175
2	Avon Wheatbelt	Nyctophilus geoffroyi				222
2	Avon Wheatbelt	Nyctophilus geoffroyi				270
2	Avon Wheatbelt	Nyctophilus geoffroyi				297
2	Avon Wheatbelt	Nyctophilus geoffroyi				310

2	Avon Wheatbelt	Nyctophilus geoffroyi	330
2	Avon Wheatbelt	Nyctophilus geoffroyi	363
2	Avon Wheatbelt	Nyctophilus geoffroyi	366
2	Avon Wheatbelt	Nyctophilus geoffroyi	368
2	Avon Wheatbelt	Nyctophilus geoffroyi	403
2	Avon Wheatbelt	Ozimops sp	245
2	Esperance Plains	Chalinolobus gouldii	333
2	Esperance Plains	Chalinolobus gouldii	482
2	Esperance Plains	Chalinolobus morio	87
2	Esperance Plains	Chalinolobus morio	120
2	Esperance Plains	Chalinolobus morio	163
2	Esperance Plains	Chalinolobus morio	234
2	Esperance Plains	Chalinolobus morio	311
2	Esperance Plains	Nyctophilus geoffroyi	357
2	Esperance Plains	Nyctophilus sp	NA
2	Esperance Plains	Vespadelus regulus	77
2	Esperance Plains	Vespadelus regulus	146
2	Esperance Plains	Vespadelus regulus	152
2	Esperance Plains	Vespadelus regulus	170
2	Esperance Plains	Vespadelus regulus	225
2	Esperance Plains	Vespadelus regulus	285
2	Esperance Plains	Vespadelus regulus	288
2	Esperance Plains	Vespadelus regulus	387
2	Esperance Plains	Vespadelus regulus	NA
2	Esperance Plains	Vespadelus regulus	NA
2	Esperance Plains	Vespadelus regulus	NA
2	Jarrah Forest	Chalinolobus gouldii	102
2	Jarrah Forest	Chalinolobus gouldii	150
2	Jarrah Forest	Chalinolobus gouldii	189
2	Jarrah Forest	Chalinolobus gouldii	200
2	Jarrah Forest	Chalinolobus gouldii	204
2	Jarrah Forest	Chalinolobus gouldii	217
2	Jarrah Forest	Chalinolobus gouldii	219
2	Jarrah Forest	Chalinolobus gouldii	230
2	Jarrah Forest	Chalinolobus gouldii	236
2	Jarrah Forest	Chalinolobus gouldii	240
2	Jarrah Forest	Chalinolobus gouldii	248
2	Jarrah Forest	Chalinolobus gouldii	264
2	Jarrah Forest	Chalinolobus gouldii	280
2	Jarrah Forest	Chalinolobus gouldii	313
2	Jarrah Forest	Chalinolobus gouldii	316
2	Jarrah Forest	Chalinolobus gouldii	317
2	Jarrah Forest	Chalinolobus gouldii	339
2	Jarrah Forest	Chalinolobus gouldii	345
2	Jarrah Forest	Chalinolobus gouldii	349
2	Jarrah Forest	Chalinolobus gouldii	349
2	Jarrah Forest	Chalinolobus gouldii	360
2	Jarrah Forest	Chalinolobus gouldii	360
2	Jarrah Forest	Chalinolobus gouldii	373
2	Jarrah Forest	Chalinolobus gouldii	416
2	Jarrah Forest	Chalinolobus gouldii	421
2	Jarrah Forest	Chalinolobus gouldii	479
2	Jarrah Forest	Chalinolobus gouldii	645
2	Jarrah Forest	Chalinolobus gouldii	711
2	Jarrah Forest	Chalinolobus gouldii	**1981**
2	Jarrah Forest	Chalinolobus gouldii	**23352**
2	Jarrah Forest	Chalinolobus morio	99
2	Jarrah Forest	Chalinolobus morio	100
2	Jarrah Forest	Chalinolobus morio	104
2	Jarrah Forest	Chalinolobus morio	121
2	Jarrah Forest	Chalinolobus morio	122
2	Jarrah Forest	Chalinolobus morio	124
2	Jarrah Forest	Chalinolobus morio	125
2	Jarrah Forest	Chalinolobus morio	130
2	Jarrah Forest	Chalinolobus morio	130
2	Jarrah Forest	Chalinolobus morio	141
2	Jarrah Forest	Chalinolobus morio	152

2	Jarrah Forest	Chalinolobus morio	153
2	Jarrah Forest	Chalinolobus morio	185
2	Jarrah Forest	Chalinolobus morio	227
2	Jarrah Forest	Chalinolobus morio	230
2	Jarrah Forest	Chalinolobus morio	235
2	Jarrah Forest	Chalinolobus morio	238
2	Jarrah Forest	Chalinolobus morio	245
2	Jarrah Forest	Chalinolobus morio	247
2	Jarrah Forest	Chalinolobus morio	255
2	Jarrah Forest	Chalinolobus morio	256
2	Jarrah Forest	Chalinolobus morio	308
2	Jarrah Forest	Chalinolobus morio	310
2	Jarrah Forest	Chalinolobus morio	317
2	Jarrah Forest	Chalinolobus morio	327
2	Jarrah Forest	Chalinolobus morio	342
2	Jarrah Forest	Chalinolobus morio	355
2	Jarrah Forest	Chalinolobus morio	409
2	Jarrah Forest	Chalinolobus morio	449
2	Jarrah Forest	Chalinolobus morio	472
2	Jarrah Forest	Chalinolobus morio	683
2	Jarrah Forest	Falsistrellus mackenziei	116
2	Jarrah Forest	Falsistrellus mackenziei	127
2	Jarrah Forest	Falsistrellus mackenziei	152
2	Jarrah Forest	Falsistrellus mackenziei	164
2	Jarrah Forest	Falsistrellus mackenziei	177
2	Jarrah Forest	Falsistrellus mackenziei	**1959**
2	Jarrah Forest	Nyctophilus geoffroyi	119
2	Jarrah Forest	Nyctophilus geoffroyi	200
2	Jarrah Forest	Nyctophilus geoffroyi	219
2	Jarrah Forest	Nyctophilus geoffroyi	221
2	Jarrah Forest	Nyctophilus geoffroyi	278
2	Jarrah Forest	Nyctophilus geoffroyi	325
2	Jarrah Forest	Nyctophilus gouldi	20
2	Jarrah Forest	Nyctophilus gouldi	91
2	Jarrah Forest	Nyctophilus gouldi	111
2	Jarrah Forest	Nyctophilus gouldi	115
2	Jarrah Forest	Nyctophilus gouldi	117
2	Jarrah Forest	Nyctophilus gouldi	145
2	Jarrah Forest	Nyctophilus gouldi	149
2	Jarrah Forest	Nyctophilus gouldi	184
2	Jarrah Forest	Nyctophilus gouldi	184
2	Jarrah Forest	Nyctophilus gouldi	189
2	Jarrah Forest	Nyctophilus gouldi	209
2	Jarrah Forest	Nyctophilus gouldi	212
2	Jarrah Forest	Nyctophilus gouldi	213
2	Jarrah Forest	Nyctophilus gouldi	222
2	Jarrah Forest	Nyctophilus gouldi	223
2	Jarrah Forest	Nyctophilus gouldi	238
2	Jarrah Forest	Nyctophilus gouldi	239
2	Jarrah Forest	Nyctophilus gouldi	242
2	Jarrah Forest	Nyctophilus gouldi	245
2	Jarrah Forest	Nyctophilus gouldi	248
2	Jarrah Forest	Nyctophilus gouldi	250
2	Jarrah Forest	Nyctophilus gouldi	266
2	Jarrah Forest	Nyctophilus gouldi	274
2	Jarrah Forest	Nyctophilus gouldi	276
2	Jarrah Forest	Nyctophilus gouldi	288
2	Jarrah Forest	Nyctophilus gouldi	288
2	Jarrah Forest	Nyctophilus gouldi	292
2	Jarrah Forest	Nyctophilus gouldi	292
2	Jarrah Forest	Nyctophilus gouldi	292
2	Jarrah Forest	Nyctophilus gouldi	311
2	Jarrah Forest	Nyctophilus gouldi	321
2	Jarrah Forest	Nyctophilus gouldi	350
2	Jarrah Forest	Nyctophilus gouldi	356
2	Jarrah Forest	Nyctophilus gouldi	360
2	Jarrah Forest	Nyctophilus gouldi	374

2	Jarrah Forest	Nyctophilus gouldi	382
2	Jarrah Forest	Nyctophilus gouldi	387
2	Jarrah Forest	Nyctophilus gouldi	389
2	Jarrah Forest	Nyctophilus gouldi	396
2	Jarrah Forest	Nyctophilus gouldi	400
2	Jarrah Forest	Nyctophilus gouldi	420
2	Jarrah Forest	Nyctophilus gouldi	440
2	Jarrah Forest	Nyctophilus gouldi	464
2	Jarrah Forest	Nyctophilus gouldi	540
2	Jarrah Forest	Nyctophilus gouldi	540
2	Jarrah Forest	Nyctophilus gouldi	563
2	Jarrah Forest	Nyctophilus gouldi	663
2	Jarrah Forest	Nyctophilus gouldi	735
2	Jarrah Forest	Nyctophilus gouldi	976
2	Jarrah Forest	Nyctophilus gouldi	**1950**
2	Jarrah Forest	Nyctophilus gouldi	NA
2	Jarrah Forest	Nyctophilus gouldi	NA
2	Jarrah Forest	Nyctophilus gouldi	NA
2	Jarrah Forest	Nyctophilus major	91
2	Jarrah Forest	Nyctophilus major	218
2	Jarrah Forest	Nyctophilus major	**1416**
2	Jarrah Forest	Nyctophilus sp	133
2	Jarrah Forest	Nyctophilus sp	146
2	Jarrah Forest	Nyctophilus sp	149
2	Jarrah Forest	Vespadelus regulus	25
2	Jarrah Forest	Vespadelus regulus	60
2	Jarrah Forest	Vespadelus regulus	65
2	Jarrah Forest	Vespadelus regulus	90
2	Jarrah Forest	Vespadelus regulus	107
2	Jarrah Forest	Vespadelus regulus	110
2	Jarrah Forest	Vespadelus regulus	111
2	Jarrah Forest	Vespadelus regulus	116
2	Jarrah Forest	Vespadelus regulus	118
2	Jarrah Forest	Vespadelus regulus	121
2	Jarrah Forest	Vespadelus regulus	123
2	Jarrah Forest	Vespadelus regulus	123
2	Jarrah Forest	Vespadelus regulus	124
2	Jarrah Forest	Vespadelus regulus	125
2	Jarrah Forest	Vespadelus regulus	126
2	Jarrah Forest	Vespadelus regulus	130
2	Jarrah Forest	Vespadelus regulus	133
2	Jarrah Forest	Vespadelus regulus	134
2	Jarrah Forest	Vespadelus regulus	134
2	Jarrah Forest	Vespadelus regulus	136
2	Jarrah Forest	Vespadelus regulus	137
2	Jarrah Forest	Vespadelus regulus	137
2	Jarrah Forest	Vespadelus regulus	137
2	Jarrah Forest	Vespadelus regulus	142
2	Jarrah Forest	Vespadelus regulus	146
2	Jarrah Forest	Vespadelus regulus	150
2	Jarrah Forest	Vespadelus regulus	154
2	Jarrah Forest	Vespadelus regulus	156
2	Jarrah Forest	Vespadelus regulus	160
2	Jarrah Forest	Vespadelus regulus	161
2	Jarrah Forest	Vespadelus regulus	162
2	Jarrah Forest	Vespadelus regulus	162
2	Jarrah Forest	Vespadelus regulus	170
2	Jarrah Forest	Vespadelus regulus	172
2	Jarrah Forest	Vespadelus regulus	178
2	Jarrah Forest	Vespadelus regulus	186
2	Jarrah Forest	Vespadelus regulus	187
2	Jarrah Forest	Vespadelus regulus	190
2	Jarrah Forest	Vespadelus regulus	194
2	Jarrah Forest	Vespadelus regulus	194
2	Jarrah Forest	Vespadelus regulus	201
2	Jarrah Forest	Vespadelus regulus	202
2	Jarrah Forest	Vespadelus regulus	202

2	Jarrah Forest	Vespadelus regulus	204
2	Jarrah Forest	Vespadelus regulus	207
2	Jarrah Forest	Vespadelus regulus	207
2	Jarrah Forest	Vespadelus regulus	216
2	Jarrah Forest	Vespadelus regulus	225
2	Jarrah Forest	Vespadelus regulus	236
2	Jarrah Forest	Vespadelus regulus	241
2	Jarrah Forest	Vespadelus regulus	241
2	Jarrah Forest	Vespadelus regulus	250
2	Jarrah Forest	Vespadelus regulus	253
2	Jarrah Forest	Vespadelus regulus	258
2	Jarrah Forest	Vespadelus regulus	261
2	Jarrah Forest	Vespadelus regulus	262
2	Jarrah Forest	Vespadelus regulus	262
2	Jarrah Forest	Vespadelus regulus	263
2	Jarrah Forest	Vespadelus regulus	266
2	Jarrah Forest	Vespadelus regulus	266
2	Jarrah Forest	Vespadelus regulus	272
2	Jarrah Forest	Vespadelus regulus	276
2	Jarrah Forest	Vespadelus regulus	279
2	Jarrah Forest	Vespadelus regulus	282
2	Jarrah Forest	Vespadelus regulus	283
2	Jarrah Forest	Vespadelus regulus	285
2	Jarrah Forest	Vespadelus regulus	287
2	Jarrah Forest	Vespadelus regulus	293
2	Jarrah Forest	Vespadelus regulus	300
2	Jarrah Forest	Vespadelus regulus	329
2	Jarrah Forest	Vespadelus regulus	340
2	Jarrah Forest	Vespadelus regulus	347
2	Jarrah Forest	Vespadelus regulus	356
2	Jarrah Forest	Vespadelus regulus	356
2	Jarrah Forest	Vespadelus regulus	364
2	Jarrah Forest	Vespadelus regulus	368
2	Jarrah Forest	Vespadelus regulus	398
2	Jarrah Forest	Vespadelus regulus	401
2	Jarrah Forest	Vespadelus regulus	406
2	Jarrah Forest	Vespadelus regulus	427
2	Jarrah Forest	Vespadelus regulus	430
2	Jarrah Forest	Vespadelus regulus	453
2	Jarrah Forest	Vespadelus regulus	457
2	Jarrah Forest	Vespadelus regulus	460
2	Jarrah Forest	Vespadelus regulus	472
2	Jarrah Forest	Vespadelus regulus	573
2	Jarrah Forest	Vespadelus regulus	595
2	Jarrah Forest	Vespadelus regulus	606
2	Jarrah Forest	Vespadelus regulus	616
2	Jarrah Forest	Vespadelus regulus	643
2	Jarrah Forest	Vespadelus regulus	717
2	Jarrah Forest	Vespadelus regulus	745
2	Jarrah Forest	Vespadelus regulus	813
2	Jarrah Forest	Vespadelus regulus	848
2	Jarrah Forest	Vespadelus regulus	947
2	Jarrah Forest	Vespadelus regulus	**1115**
2	Jarrah Forest	Vespadelus regulus	**1128**
2	Jarrah Forest	Vespadelus regulus	**1149**
2	Jarrah Forest	Vespadelus regulus	**1818**
2	Jarrah Forest	Vespadelus regulus	NA
2	Jarrah Forest	Vespadelus regulus	NA
2	Jarrah Forest	Vespadelus sp	517
2	Swan Coastal Plain	Chalinolobus gouldii	179
2	Swan Coastal Plain	Chalinolobus gouldii	247
2	Warren	Chalinolobus morio	182
2	Warren	Chalinolobus morio	204
2	Warren	Chalinolobus morio	242
2	Warren	Chalinolobus morio	261
2	Warren	Chalinolobus morio	281
2	Warren	Chalinolobus morio	333

2	Warren	Chalinolobus morio	356
2	Warren	Chalinolobus morio	359
2	Warren	Chalinolobus morio	369
2	Warren	Chalinolobus morio	375
2	Warren	Chalinolobus morio	386
2	Warren	Chalinolobus morio	388
2	Warren	Chalinolobus morio	395
2	Warren	Chalinolobus morio	397
2	Warren	Chalinolobus morio	419
2	Warren	Chalinolobus morio	447
2	Warren	Chalinolobus morio	452
2	Warren	Chalinolobus morio	582
2	Warren	Chalinolobus morio	724
2	Warren	Chalinolobus morio	753
2	Warren	Chalinolobus morio	**1166**
2	Warren	Chalinolobus morio	**1645**
2	Warren	Chalinolobus morio	**2602**
2	Warren	Chalinolobus morio	NA
2	Warren	Chalinolobus morio	NA
2	Warren	Falsistrellus mackenziei	928
2	Warren	Nyctophilus geoffroyi	223
2	Warren	Nyctophilus geoffroyi	447
2	Warren	Nyctophilus gouldi	138
2	Warren	Nyctophilus gouldi	233
2	Warren	Nyctophilus gouldi	249
2	Warren	Nyctophilus gouldi	280
2	Warren	Nyctophilus gouldi	285
2	Warren	Nyctophilus gouldi	309
2	Warren	Nyctophilus gouldi	334
2	Warren	Nyctophilus gouldi	348
2	Warren	Nyctophilus gouldi	358
2	Warren	Nyctophilus gouldi	403
2	Warren	Nyctophilus gouldi	694
2	Warren	Nyctophilus gouldi	783
2	Warren	Nyctophilus gouldi	884
2	Warren	Nyctophilus gouldi	**1229**
2	Warren	Nyctophilus gouldi	**1544**
2	Warren	Nyctophilus major	258
2	Warren	Nyctophilus sp	273
2	Warren	Vespadelus regulus	0
2	Warren	Vespadelus regulus	167
2	Warren	Vespadelus regulus	192
2	Warren	Vespadelus regulus	207
2	Warren	Vespadelus regulus	213
2	Warren	Vespadelus regulus	226
2	Warren	Vespadelus regulus	235
2	Warren	Vespadelus regulus	238
2	Warren	Vespadelus regulus	243
2	Warren	Vespadelus regulus	250
2	Warren	Vespadelus regulus	257
2	Warren	Vespadelus regulus	264
2	Warren	Vespadelus regulus	274
2	Warren	Vespadelus regulus	292
2	Warren	Vespadelus regulus	295
2	Warren	Vespadelus regulus	295
2	Warren	Vespadelus regulus	300
2	Warren	Vespadelus regulus	301
2	Warren	Vespadelus regulus	308
2	Warren	Vespadelus regulus	310
2	Warren	Vespadelus regulus	318
2	Warren	Vespadelus regulus	320
2	Warren	Vespadelus regulus	320
2	Warren	Vespadelus regulus	342
2	Warren	Vespadelus regulus	351
2	Warren	Vespadelus regulus	358
2	Warren	Vespadelus regulus	358
2	Warren	Vespadelus regulus	392

2	Warren	Vespadelus regulus	393
2	Warren	Vespadelus regulus	421
2	Warren	Vespadelus regulus	431
2	Warren	Vespadelus regulus	462
2	Warren	Vespadelus regulus	464
2	Warren	Vespadelus regulus	497
2	Warren	Vespadelus regulus	849
2	Warren	Vespadelus regulus	**1026**
2	Warren	Vespadelus regulus	**1054**
2	Warren	Vespadelus regulus	**1187**
2	Warren	Vespadelus regulus	**1192**
2	Warren	Vespadelus regulus	**2678**
2	Warren	Vespadelus regulus	NA

*Samples collected at either the Jarrah Forest or the Swan Coastal Plain Bioregions
NA, assay failed.
